# Intraocular Gnathostomiasis Manifested as a Unilateral Panuveitis

**DOI:** 10.7759/cureus.95886

**Published:** 2025-11-01

**Authors:** Chansathya Khieu, Kosal Thay, Monineat Sim, Meng Ngy

**Affiliations:** 1 Department of Ophthalmology, Khmer-Soviet Friendship Hospital, Phnom Penh, KHM; 2 Department of Ophthalmology, Meng-Rutnin Eye Specialists, Phnom Penh, KHM; 3 Department of Medical Laboratory, Khmer-Soviet Friendship Hospital, Phnom Penh, KHM

**Keywords:** anterior uveitis, gnathostoma spinigerum, intraocular gnathostomiasis, secondary glaucoma, vitreous hemorrhage

## Abstract

A 50-year-old Cambodian man presented with unilateral blurred vision that developed shortly after an ischemic stroke. Initial ocular examination showed anterior uveitis, mild retinal vasculopathy with vitreous hemorrhage. Two days later, a live white worm was observed embedded in the iris, migrating through the iris tissue and leaving multiple tracks, causing increased intraocular pressure and secondary glaucoma. A pre-operative diagnosis of intraocular parasitic infection was made, and the worm was surgically extracted. The specimen was identified as *Gnathostoma spinigerum,* and eventually this was diagnosed as intraocular gnathostomiasis.

## Introduction

Gnathostomiasis is a food-borne zoonotic nematode infection caused by *Gnathostoma spinigerum*. It is most prevalent in developing countries and was first reported in Cambodia in 2015. Human infection is typically associated with the consumption of raw or undercooked freshwater fish and crustaceans. Intraocular involvement is rare but potentially vision-threatening [1].

## Case presentation

A 50-year-old Cambodian man was referred to our ophthalmology clinic with a one-week history of blurred vision in the left eye (LE). His past medical history was significant for hypertension, dyslipidemia, leukocytosis, and a recent ischemic stroke for which he had been treated at another center.

**Figure 1:**
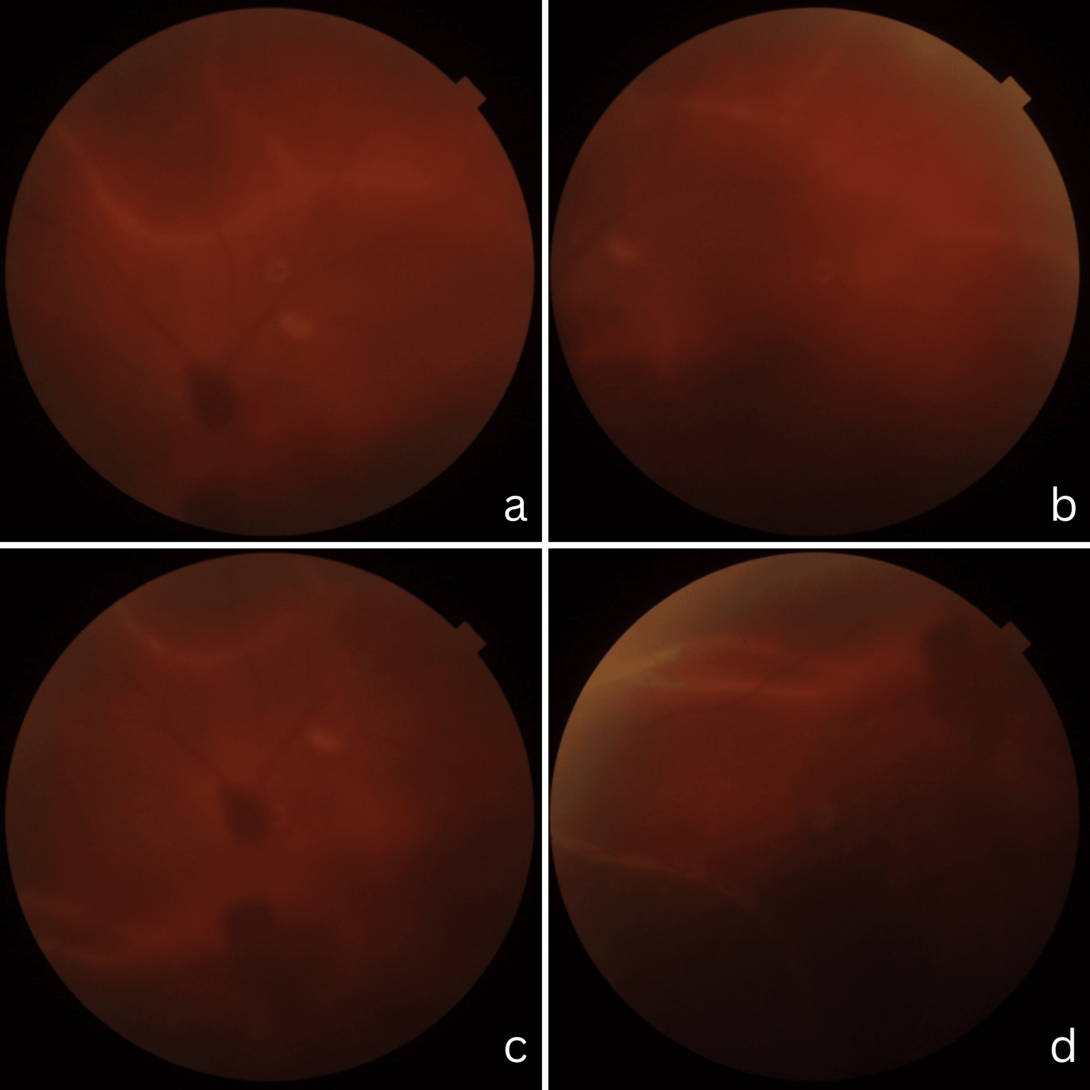
Fundus of the left eye presenting white streak and vitreous opacity a, b, c, and d show vitreous opacity and hemorrhage with multiple white streak across the posterior pole of left retina

On examination, best-corrected visual acuity was 6/7.5 in the right eye (RE) and hand motion (HM) in the LE, with normal intraocular pressure (IOP) in both eyes. Anterior and posterior segment evaluation of the RE was unremarkable. In the LE, slit-lamp examination revealed 1+ anterior chamber (AC) cells according to the Standardization of Uveitis Nomenclature (SUN) classification [2], scattered pigment cells, and cataract. Posterior segment examination LE revealed dense vitreous opacities with possible vitreous hemorrhage, scattered retinal hemorrhages, and whitish subretinal streak-like lesions (Figure 1). These findings were not typical of classic hypertensive retinopathy (usually bilateral) [3], although the patient’s recent stroke suggested a vascular etiology.

**Figure 2:**
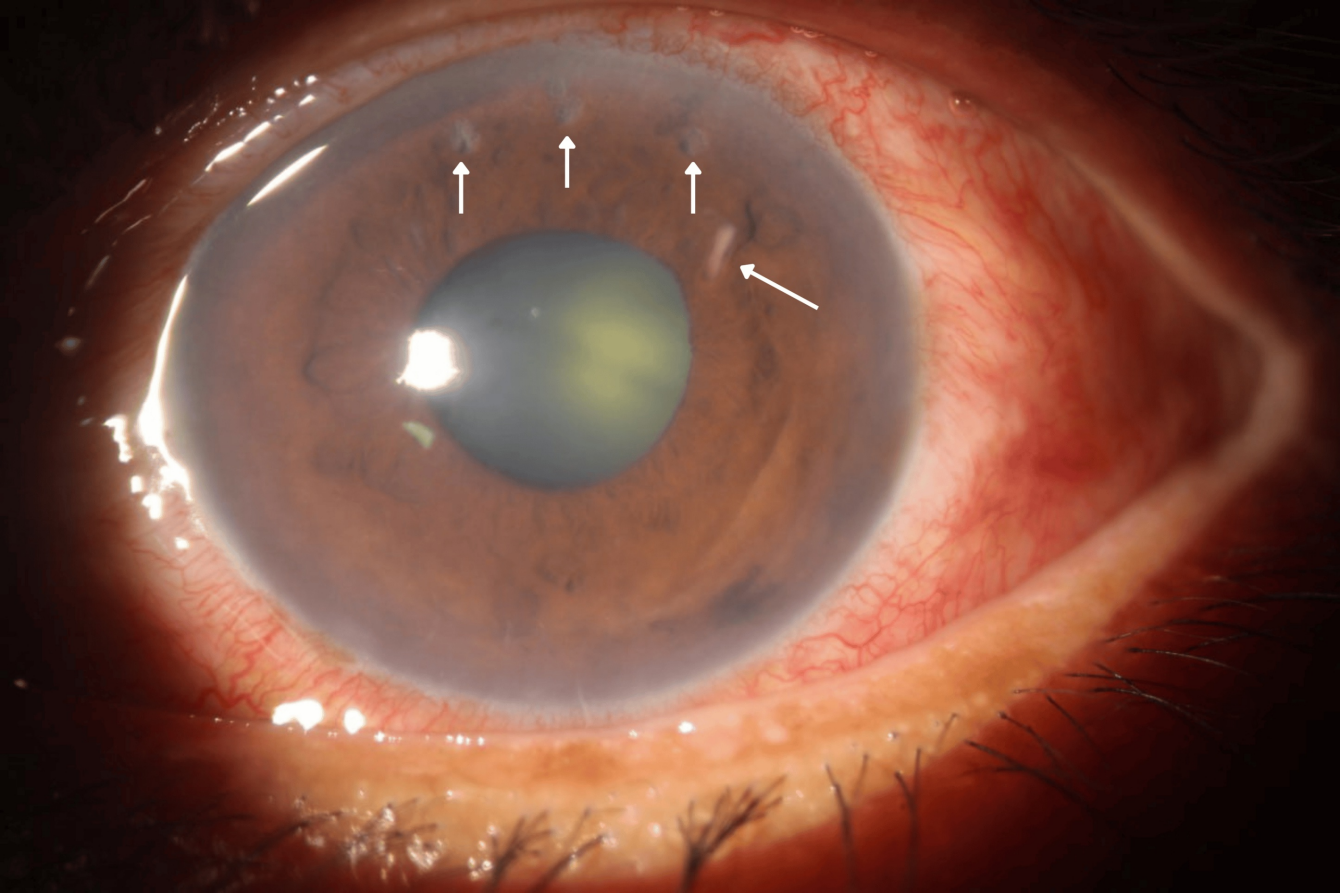
White worm-like lesion and iris holes (day 1)

**Figure 3:**
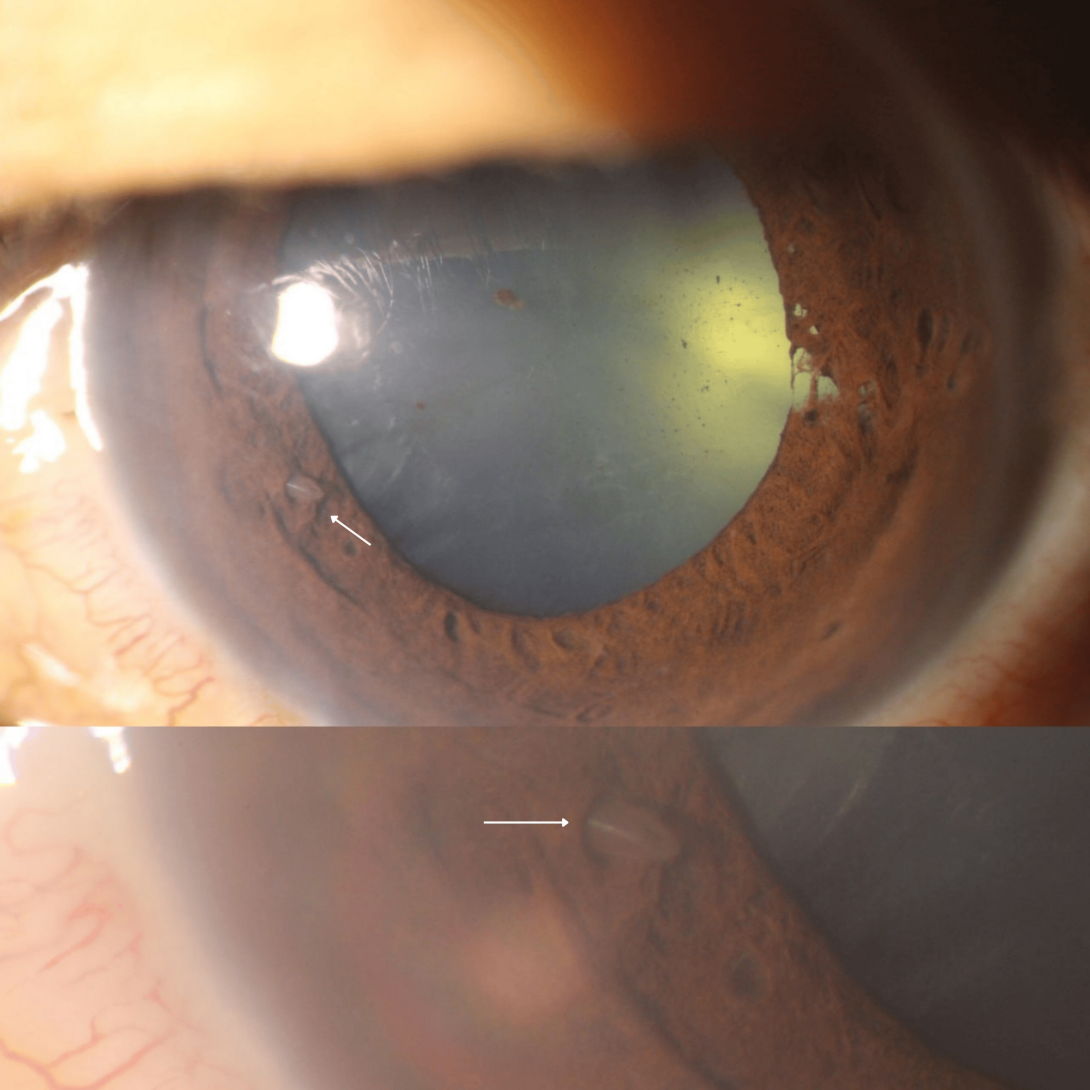
Presentation of parasite stuck in iris, shifting location pre-operation (day 2) a shows the left eye with a dilated pupil, traumatic iris, and cataract formation. b shows a zoom-in photo of the left eye anterior segment, focusing on a parasite embedded in the iris

A presumptive diagnosis of retinal vasculopathy complicated by vitreous hemorrhage was initially made, and the patient was referred for systemic evaluation. One day later, he returned with severe bulging pain in the LE. Slit-lamp examination revealed a motile white worm embedded in the iris, along with three iris perforations (Figure 2). Intraocular pressure (IOP) was 36 mmHg. After IOP-lowering therapy, the worm was no longer visible. The posterior segment remained obscured by media opacities. At the following day’s review, IOP had decreased to 30 mmHg under antiglaucoma medication, pain was reduced, but vision remained blurred. A new iris defect was noted, and the worm was visualized at a different site, confirming the diagnosis of intraocular parasitic infection (Figure 3).

Surgical intervention was performed the same day to prevent further migration. Under retrobulbar anesthesia, an anterior chamber paracentesis was made, and a viscoelastic surgical device (VSD) was injected, but the worm had migrated out of view. Given the coexisting visually significant cataract, a routine temporal approach phacoemulsification without intraocular lens implantation was performed to improve visualization. During cortical removal, the worm was discovered lodged between the posterior capsule and the anterior hyaloid. The capsule was punctured, and the worm was successfully extracted using retinal microforceps. The specimen was sent for parasitological identification.

**Figure 4:**
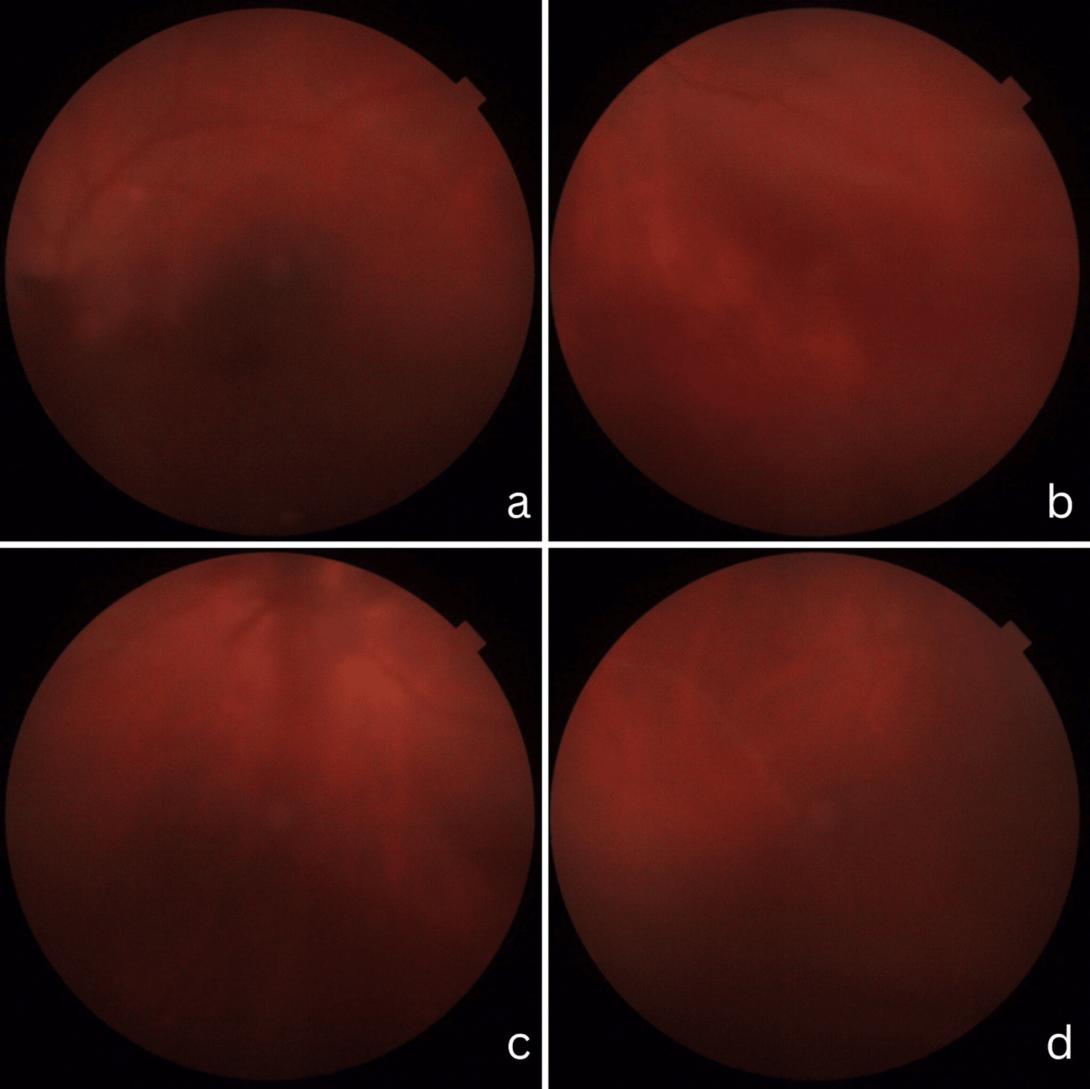
Fundus of left eye, three weeks post-operatively along with anti-parasite and anti-inflammation treatment a, b, c, and d show the left fundus photograph post-operatively with less vitreous opacity and disappearance of white streaks across the retina

At three weeks post-operatively, vision in the LE improved from hand motion to 6/30 with +10.00 D aphakic correction. The anterior chamber was quiet, vitreous opacities had decreased, and the subretinal whitish streaks had largely resolved (Figure 4).

Laboratory diagnosis and treatment

**Figure 5:**
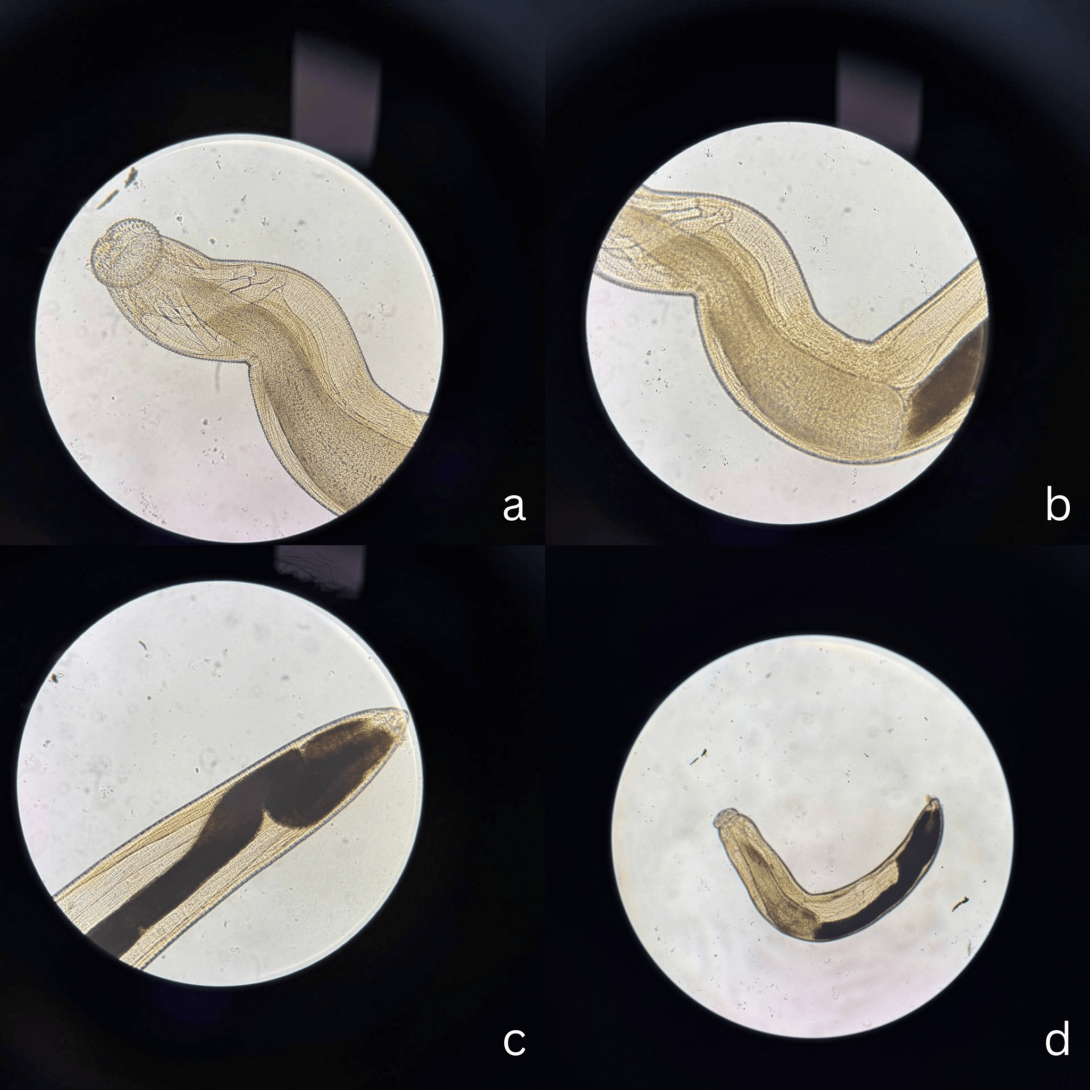
Microscopic image of Gnathostoma spinigerum a, b, and c show sectoral cuts of *Gnathostoma spp.* d shows the full image of parasite by 4x objective lens microscope

Direct microscopic examination revealed a reddish-white nematode measuring 3.5 mm in length and 0.3 mm in width, with a characteristic cephalic bulb bearing four rows of hooklets and two pairs of cervical spines (Figure 5). Morphological features were consistent with a third-stage larva of *Gnathostoma spinigerum*. Post-operatively, the patient was treated with oral albendazole 400 mg twice daily for 21 days [4], systemic corticosteroids (prednisolone, initial dose 30 mg daily, tapered gradually), topical post-operative medications, and continued antiglaucoma therapy.

## Discussion

Ocular gnathostomiasis is a rare manifestation of gnathostomiasis, most frequently reported in South and Southeast Asia [5]. A review by Nawa et al. identified more than 80 ocular cases worldwide [6]. In Cambodia, the first cases were documented in 2015, presenting as anterior uveitis and neuroretinitis [1,7]. Reported ocular manifestations range from anterior segment inflammation to orbital cellulitis, posterior uveitis, retinal vascular occlusion, vitreous hemorrhage, and even retinal detachment [8,9].

In our patient, the initial presentation mimicked retinal vasculopathy related to a recent ischemic stroke. Only later, with direct visualization of a motile worm in the iris, was intraocular gnathostomiasis confirmed. This underscores the importance of careful clinical assessment and maintaining suspicion in atypical cases.

Management involves prompt surgical extraction of the parasite, followed by systemic anti-parasitic therapy (e.g. albendazole or ivermectin) and corticosteroids to control inflammation [10]. Early diagnosis and intervention are critical to prevent irreversible ocular damage.

## Conclusions

Intraocular gnathostomiasis is rare but treatable. Early recognition through meticulous examination and direct parasite identification is essential for preserving vision. From a public health perspective, education on the risks of consuming raw or undercooked freshwater fish remains key to prevention.
